# Differential regulation of soil microecology in crop rotation systems of maize, seed pumpkin, and processing tomato

**DOI:** 10.3389/fmicb.2025.1640980

**Published:** 2025-11-17

**Authors:** Xingxing Liu, Xuyuan Li, Menglei Feng, Xuliang Liu, Xiaoyu Zhu, Yulong Zhang, Ge Zhang, Aiying Wang

**Affiliations:** 1 College of Life Sciences, Shihezi University, Shihezi, Xinjiang, China; 2 Key Laboratory of Oasis Town and Mountain-Basin System Ecology, Xinjiang Production and Construction Corps, Shihezi, Xinjiang, China; 3 Xinjiang Greel Agricultural Technology Co., Ltd, Shihezi, Xinjiang, China

**Keywords:** processing tomato, crop rotation, enzyme activity, microbial community diversity, microbial co-occurrence network

## Abstract

Long-term continuous cropping of processing tomatoes in Xinjiang has led to soil degradation and microecological imbalance, severely constraining the sustainable development of the industry. To investigate the mitigation mechanisms of different crop rotation systems, this study established maize-tomato rotation (SZa), pumpkin (for seeds)-tomato rotation (SLa), and continuous cropping control (SSa) treatments in a long-term continuously cropped tomato field. The results demonstrated that compared to SSa, the SLa treatment increased the proportion of large aggregates (>2 mm) by 16.5%, whereas the SZa treatment decreased it by 24.6%. Rotation significantly reduced soil pH (by 5.6%−6.0%) and increased electrical conductivity (by 124%−215%). Enzyme activities responded variably: phosphatase activity increased by 13.9%, while urease and sucrase activities significantly decreased. Microbial α-diversity was significantly enhanced, with the Shannon index for bacteria and fungi increasing by up to 10.3% and 24.3%, respectively. Network analysis revealed that SZa optimized bacterial network complexity, while SLa specifically reduced the abundance of Ascomycota (by 17.5%) and reshaped the fungal community. Notably, the SLa treatment significantly decreased soil total potassium content by 13.6%. This study confirms that both maize and pumpkin rotation can regulate the soil microecology through differentiated strategies, providing an important theoretical basis for optimizing cropping systems of processing tomatoes in Xinjiang.

## Graphical Abstract

**Figure:**
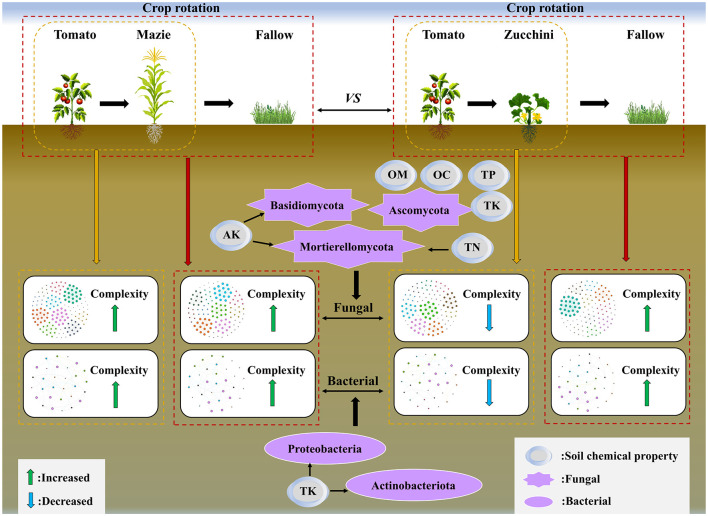
Illustration of crop rotation effects on soil health, comparing tomato-maize-fallow with tomato-zucchini-fallow cycles. It highlights fungal groups (Basidiomycota, Ascomycota, Mortierellomycota) and bacterial groups (Proteobacteria, Actinobacteriota) along with soil chemical properties (OM, OC, TP, TK, TN, AK). Complexity changes for fungal and bacterial communities are indicated, showing increases or decreases with arrows.

## 1 Introduction

Xinjiang has emerged as a globally significant tomato production base, with the industry serving as a vital component of the regional agricultural economy due to its unique geographical advantages. The region benefits from exceptional solar radiation, thermal resources, and marked diurnal temperature variation, which contribute to premium tomato quality and strong international competitiveness (Jia et al., 2023). Processing tomatoes dominate local production, comprising over 80% of total output, making this sector critically important for regional economic development (Zhou et al., 2024).

However, this agricultural success faces sustainability challenges from long-term monoculture practices. Continuous processing tomato cultivation has caused severe soil degradation and ecological imbalance, deteriorating soil's physical, chemical, and biological properties (Guo et al., 2024). The disruption of soil microbial communities represents a primary concern (Shang et al., 2023), where suppression of beneficial microorganisms compromises natural pathogen control and nutrient cycling functions (Agbede and Oyewumi, 2022; Liu et al., 2022c). Concurrently, excessive depletion of essential nutrients (N, P, K, and micronutrients) has diminished soil fertility, directly impairing crop productivity and threatening long-term industry viability (Han et al., 2022; Ku et al., 2022).

The complex diversity of soil microbiomes plays fundamental roles in maintaining soil health and fertility (Fan et al., 2022). Crop rotation has demonstrated significant potential in mitigating monoculture impacts through multiple mechanisms: diversified root exudates enhance microbial biodiversity and functional redundancy (Li et al., 2021); disruption of pathogen life cycles reduces disease pressure; and improved organic matter decomposition optimizes nutrient retention (Su et al., 2022). The maize-soybean intercropping system exemplifies these benefits, where rhizosphere interactions increase microbial network complexity and ecosystem stability—contrasting sharply with the ecological simplification under continuous tomato cultivation (Liu et al., 2022a; Liu and Zhao, 2023).

As a cornerstone of sustainable agriculture, strategic crop rotation addresses global soil degradation challenges by preventing nutrient depletion through diversified cropping sequences while enhancing microbial carbon sources via species-specific root exudates. This practice maintains soil porosity and organic matter dynamics, representing an essential approach for conserving farmland ecosystems and ensuring the sustainable future of Xinjiang's processing tomato industry. Properly designed rotation systems can effectively balance agricultural productivity with ecological resilience in the region.

To achieve agricultural sustainability, Xinjiang's processing tomato industry must optimize cropping systems to ensure long-term stability (Gamage et al., 2024). Crop rotation effectively alleviates monoculture limitations, enhances soil fertility, and promotes ecological resilience for sustainable production (Khan and Bhatt, 2023; Diatta et al., 2024). The maize-based rotation system introduces additional biodiversity into agroecosystems, a widely adopted global practice (Dialameh and Ghane, 2023; Li et al., 2024). As a pivotal rotation crop, maize improves soil health and crop productivity by enhancing soil structure and increasing organic matter content (Tang et al., 2022; Niu et al., 2024). Although relatively unconventional agriculturally (Abd-Elkader et al., 2022), seed pumpkin shows significant rotation potential through preliminary studies, demonstrating particular suitability for diversified systems.

This study investigated the effects of crop rotation systems involving maize, seed pumpkin, and processing tomatoes on soil microecology to provide a scientific basis for optimizing tomato cultivation practices. Specifically, we (a) examined the impacts of rotating processing tomatoes with these two crops on soil chemical properties and enzyme activities, (b) assessed microbial community diversity, composition, and co-occurrence network responses to the rotation systems, and (c) identified key soil factors driving microbial community dynamics in these cropping systems.

## 2 Materials and methods

### 2.1 Experimental site

The experiment was conducted at the experimental station of Shihezi University (44°20′N, 85°50′E) in Shihezi City, Xinjiang Uygur Autonomous Region. The site features flat terrain and is representative of a typical oasis irrigated agricultural zone, surrounded by contiguous farmland and distanced from residential areas, industrial zones, and major transportation routes, resulting in minimal human disturbance. The region experiences a temperate continental plateau climate, with the highest temperatures occurring in July, averaging between 25.2 °C and 26.2 °C and reaching a maximum of 42.2 °C. The lowest temperatures are observed in January, averaging between −18.6 °C and −15.5 °C, with extremes dropping to −37.8 °C. The mean annual precipitation is 213 mm, while the annual evaporation reaches 1,537 mm. The experimental field supports only a single growing season per year, with no winter cropping following the harvest.

### 2.2 Experimental design

The experiment employed a completely randomized block design, comprising six treatments resulting from the combination of three cropping patterns and two sampling time points, with three replicates per treatment. The study was conducted on a field that had been monocropped with processing tomatoes for 11 consecutive years. In the 12th year, all plots were randomly assigned to one of three cropping patterns: continuous monocropping of processing tomatoes (*Solanum lycopersicum* L.), rotation of processing tomatoes with maize (*Zea mays* L.), and rotation of processing tomatoes with seed pumpkin (*Cucurbita pepo* L.). All treatment plots were spatially arranged concurrently within the same year.

Soil samples were collected following the rotation phase (post-harvest in 2022) and after a subsequent fallow year (same period in 2023). The six treatment combinations were: continuous monocropping of processing tomatoes sampled after the rotation season (SSa), tomato-maize rotation sampled after rotation (SZa), tomato-pumpkin rotation sampled after rotation (SLa), continuous monocropping of processing tomatoes sampled after one fallow year (SSAFa), tomato-maize rotation sampled after one fallow year (SZAFa), and tomato-pumpkin rotation sampled after one fallow year (SLAFa). This design facilitates the separate analysis of the independent and interactive effects of cropping patterns and fallow practices on soil properties.

### 2.3 Soil sampling and analysis

Plot delineation and isolation: All treatments were established within a single large experimental field characterized by uniform soil fertility. A completely randomized block design was employed, with each individual plot measuring 2 m × 5 m. Isolation rows 0.5 m wide were established between plots of different treatments to prevent interference from agricultural practices such as irrigation and fertilization, and to ensure no intermingling of root systems occurred. Sufficient distance was also maintained between replicated blocks to avoid spatial autocorrelation. This design ensured that any observed differences in soil properties were most likely attributable to treatment effects rather than to inherent spatial heterogeneity of the soil background.

Soil sampling methodology: To maximally represent the soil conditions within each plot, soil samples were collected using a five-point sampling method (four corners and the center) from each plot. Sampling was conducted post-harvest, specifically collecting soil from the 5–15 cm rhizosphere depth layer for subsequent physico-chemical analysis. The five sub-samples from each plot were thoroughly homogenized to form one composite replicate sample. This process was repeated for three biological replicates per treatment. Samples were stored at ambient temperature post-collection. Samples designated for assessing the immediate effects of rotation (SSa, SZa, SLa) were collected after the crop harvest in 2022. Samples for evaluating the subsequent fallow effect (SSAFa, SZAFa, SLAFa) were collected at the corresponding time in 2023 to ensure temporal consistency between sampling years (see Supplementary Figure S1).

Soil aggregate structure analysis was conducted using a combined dry-wet sieving protocol. Air-dried soil samples (200 g) were subjected to dry sieving through a nested sieve set (2–0.25 mm) with manual horizontal-vertical oscillation for 2 min, followed by weighing of aggregate fractions retained on each sieve. Subsamples (50 g) from dry-sieved fractions were then analyzed via wet sieving using a TPE-100 aggregate analyzer (Zhejiang Top Cloud-Agri Technology), incorporating programmed mechanical vibration and simulated rainfall application for 30 min to isolate water-stable aggregates. Aggregate size distribution was calculated based on total dry mass and wet-sieved subsample weights, quantifying the impacts of mechanical and hydrological dispersion forces on aggregate stabilization. This standardized methodology enables precise evaluation of both physical stability and water-resistant structural integrity in cultivated soils.

Soil enzyme activity was determined using colorimetry. In soil agrichemical analysis, soil electrical conductivity (EC) and pH were measured using the water extraction method. Soil organic matter or organic carbon content was assessed via the dichromate titration method, and total nitrogen content was determined using the high—chlorine acid—sulfuric acid digestion method. Total phosphorus was measured by acid solubilization—molybdenum—antimony—arsenic colorimetry, and total potassium was determined by acid solubilization—atomic absorption spectroscopy. Nitrate and ammonium nitrogen were extracted with calcium chloride solution for determination, available phosphorus was measured using the sodium bicarbonate extraction—molybdenum—antimony—arsenic colorimetric method, and available potassium was determined by ammonium acetate extraction—atomic absorption spectroscopy (Han et al., 2024).

### 2.4 Microbial DNA sequencing

Genomic DNA of rhizosphere soil microorganisms was extracted using the DNeasy PowerSoil Pro Kit (Qiagen, Germany), with negative extraction controls included to monitor potential contamination. Amplicon sequencing was performed by Novogene Co., Ltd. (Beijing, China). The V3-V4 hypervariable region of the bacterial 16S rRNA gene was amplified using primers 338F/806R, and the ITS1 region of fungi was amplified using primers ITS1F/ITS2. The PCR reaction mixture (25 μL) consisted of 12.5 μL of 2 × KAPA HiFi HotStart ReadyMix, 1 μM of each forward and reverse primer, and approximately 20 ng of template DNA. The thermal cycling conditions were as follows: initial denaturation at 95 °C for 3 min; followed by 25–35 cycles of denaturation at 95 °C for 30 s, annealing at 55 °C for 30 s, and extension at 72 °C for 30 s; with a final extension at 72 °C for 5 min. PCR negative controls were also included. The resulting amplicons were purified, quantified, and subjected to paired-end (PE250) sequencing on an Illumina NovaSeq 6000 platform.

Raw sequencing data were processed using the QIIME 2 pipeline (version 2023.9). Briefly, quality filtering, denoising, and generation of an amplicon sequence variants (ASVs) table were performed via the q2-dada2 plugin. DADA2 employs a machine learning-based error model to correct sequencing errors. For 16S data, sequence truncation parameters were applied, while for ITS data, quality filtering was enabled while retaining length variability. Subsequently, contaminants identified from the negative controls were identified and removed. Finally, taxonomic assignment of representative sequences was conducted using the q2-feature-classifier plugin against the SILVA database (v138) for bacteria and the UNITE database (v9.0) for fungi.

### 2.5 Statistical analysis

A three-way completely randomized analysis of variance (3-way ANOVA) was employed to assess the main and interactive effects of “crop type (maize vs. seed pumpkin) × rotation pattern (monocropping vs. rotation) × fallowing (with vs. without)” on 11 soil chemical indices (OM, OC, TN, ${\text{NO}}_{3}^{+}$-N, ${\text{NH}}_{4}^{+}$-N, TP, AP, TK, AK, pH, EC) and the activities of 4 enzymes (phosphatase, urease, dehydrogenase, sucrase). All *post hoc* multiple comparisons were conducted using Tukey's HSD test, with a uniform significance threshold set at ^*^p^*^ <0.05.

Microbial community α-diversity was assessed using the Shannon, Chao1, and Pielou indices. β-diversity was evaluated based on weighted UniFrac distances and visualized via principal coordinate analysis (PCoA). The associations between community dissimilarities and soil variables were assessed using Mantel tests, with results considered significant at ^*^p^*^ <0.05.

Network analysis was performed based on 16S and ITS sequencing data. The data were uniformly pre-processed by removing rare OTUs with a detection rate of <20%. Spearman's rank correlation was then used to calculate the correlation coefficient (ρ) between OTUs. After Benjamini-Hochberg correction, only positive correlations with |ρ| ≥ 0.6 and a corrected ^*^p^*^ ≤ 0.001 were retained to exclude false positives and highlight high-confidence interactions. Undirected weighted networks were constructed using the igraph package (in R), with self-loops and duplicate edges removed. The Fast-Greedy (Clauset-Newman-Moore) algorithm was applied to maximize the modularity (Q value) for identifying potential functional sub-communities. Node sizes were scaled according to degree centrality, and modules were distinguished by color. The final networks were exported in.graphML format and visualized using Gephi 0.10.1 for layout optimization, aiming to reveal the effects of rotation and fallowing on microbial interaction structures.

Statistical analyses were conducted based on the factors in the experimental design. To separately elucidate the effects of rotation patterns and fallowing on indicators such as soil aggregates, chemical properties, and enzyme activities, the data were analyzed in grouped comparisons. One-way ANOVA followed by Tukey's *post hoc* test was performed on the non-fallow treatments (SSa, SZa, SLa) to assess the immediate effects of different rotation patterns. The same analysis was conducted on the fallow treatments (SSAFa, SZAFa, SLAFa) to evaluate the impacts of different preceding crops on the soil after 1 year of fallowing.

To evaluate the detection sensitivity of the current sample size for key differences, a *post hoc* power analysis was performed for four core metrics—bacterial Shannon index, bacterial Chao1 index, fungal Shannon index, and fungal Chao1 index—using IBM SPSS SamplePower. Under the setting of an independent samples *t*-test (α = 0.05, *n* = 3 per group), the statistical power for the “rotation vs. continuous cropping” comparison (SZa vs. SSa) was approximately 0.71. Comparisons between other groups yielded similar values. Although these power values indicate a certain detection capability, they remain below the conventional threshold of 0.80. This suggests that future studies aiming to detect medium or smaller effect sizes might consider increasing the number of replicates to enhance statistical power.

## 3 Results

### 3.1 Soil chemical properties and enzyme activities

**Figure 1:**
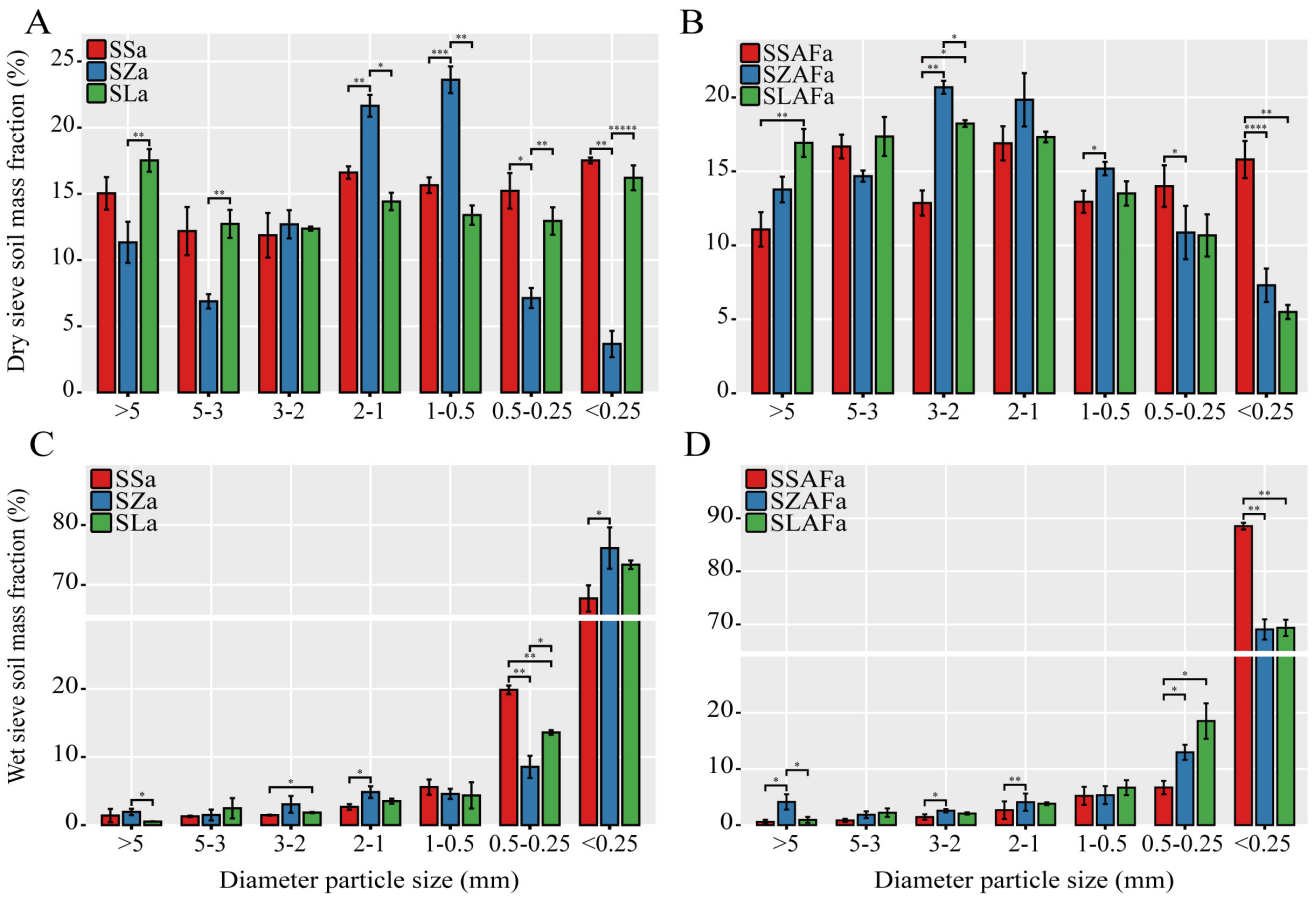
Soil aggregate size distribution under different crop rotation systems: dry sieving analysis **(A, B)** and wet sieving analysis **(C, D)**. Significant differences are indicated as *p < 0.05, **p < 0.01, and ***p < 0.001 vs. the continuous monoculture control.

Dry sieving analysis revealed that SSa and SZa treatments significantly altered the aggregate size distribution. Compared with SSa, the SZa treatment increased the proportion of small aggregates (0.25–2 mm) from 52.3 ± 1.1% to 58.9 ± 1.4% (*p* = 0.009), while simultaneously decreasing the proportions of large aggregates (>2 mm) from 15.04 ± 1.23% to 11.48 ± 0.89% (*p* = 0.011) and micro-aggregates (<0.25 mm) from 17.56 ± 0.21% to 14.66 ± 0.67% (*p* = 0.018) (Figure 1A). Compared with SSAFa, both SZAFa and SLAFa treatments significantly increased the proportion of large aggregates, from 13.9 ± 0.9% to 18.2 ± 1.1% (SZAFa, *p* = 0.002) and 19.5 ± 0.8% (SLAFa, *p* = 0.001), respectively, while reducing the proportion of micro-aggregates to 12.3 ± 0.5% (SZAFa, *p* = 0.012) and 11.8 ± 0.7% (SLAFa, *p* = 0.009), respectively (Figure 1B).

Wet sieving results demonstrated that SZa and SLa treatments significantly reduced the proportion of small aggregates compared to SSa, from 48.2 ± 1.0% to 42.1 ± 0.8% (SZa, *p* = 0.008) and 40.5 ± 1.2% (SLa, *p* = 0.005), respectively. Concurrently, both treatments increased the proportion of micro-aggregates: SZa from 20.1 ± 0.6% to 25.3 ± 0.9% (*p* = 0.003), and SLa to 27.1 ± 1.0% (*p* = 0.001) (Figure 1C). The SZAFa treatment significantly increased the proportion of large aggregates compared to SSAFa, from 15.1 ± 0.7% to 19.8 ± 0.9% (*p* = 0.002). Both SZAFa and SLAFa treatments significantly reduced the micro-aggregate content to 13.9 ± 0.6% (*p* = 0.011) and 13.2 ± 0.8% (*p* = 0.007), respectively (Figure 1D). These findings indicate that the rotation-fallow system facilitates the optimization of water-stable aggregate structure in soil.

**Figure 2:**
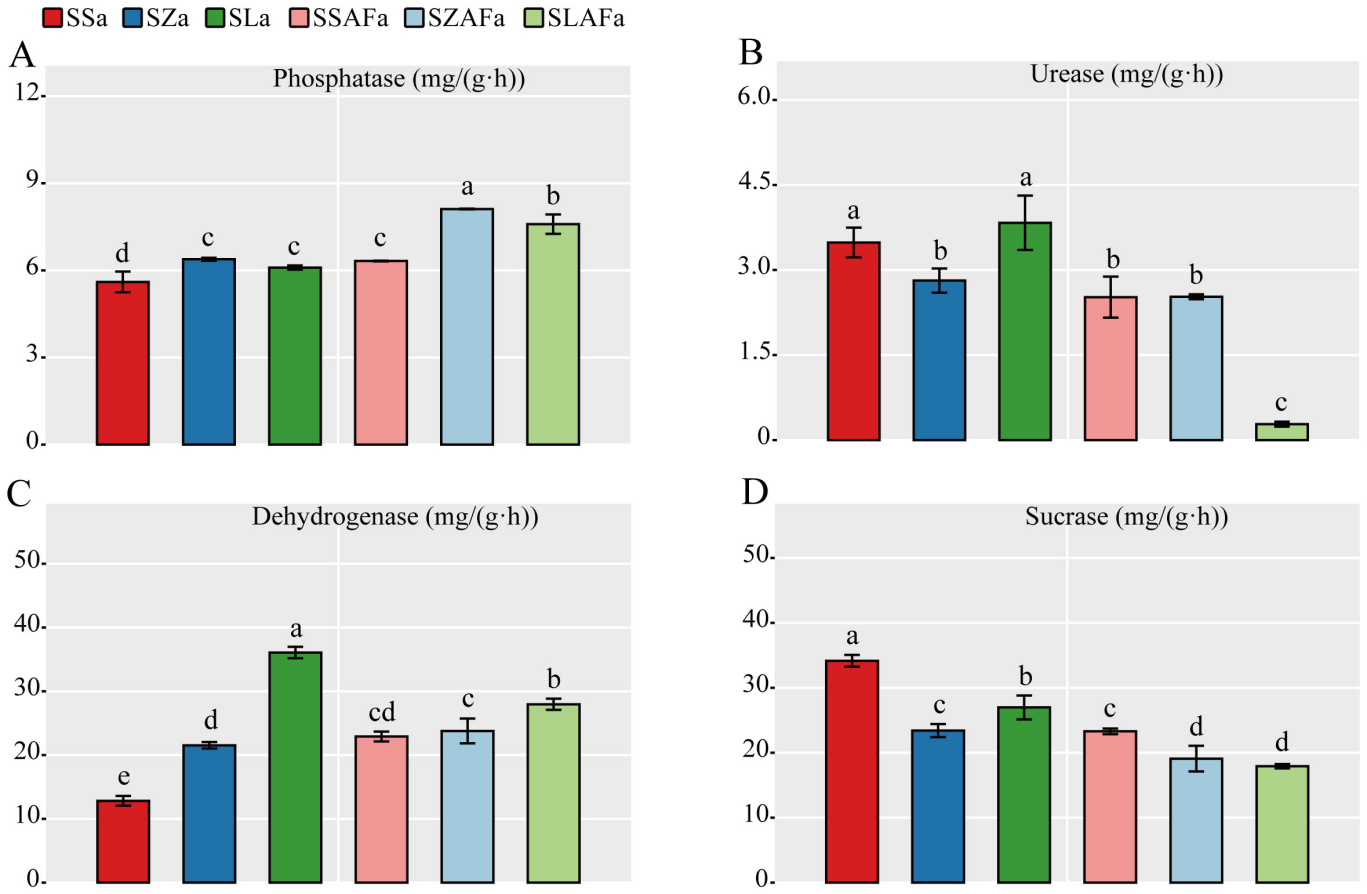
Soil phosphatase activity **(A)**, urease activity **(B)**, dehydrogenase activity **(C)**, and invertase activity **(D)** under different crop rotation patterns.

Enzyme activity analysis demonstrated significant treatment effects on soil biochemical properties. Compared to the continuous cropping control (SSa), both rotation treatments (SZa and SLa) significantly enhanced phosphatase activity, with SZa increasing from 5.60 ± 0.36 to 6.38 ± 0.05 mg g^−1^ h^−1^ (*p* = 0.004) and SLa to 6.10 ± 0.08 mg g^−1^ h^−1^ (*p* = 0.032) (Figure 2A). This enhancing effect was further amplified after fallow treatment, where SZAFa increased from 6.32 ± 0.02 to 8.11 ± 0.01 mg g^−1^ h^−1^ (*p* = 0.003) and SLAFa to 7.93 ± 0.04 mg g^−1^ h^−1^ (*p* = 0.005) compared to SSAFa. In contrast, urease activity was significantly inhibited by SZa treatment (from 3.48 ± 0.26 to 2.81 ± 0.22 mg g^−1^ h^−1^, *p* = 0.007) and particularly by SLAFa treatment (from 2.88 ± 0.03 to 0.27 ± 0.04 mg g^−1^ h^−1^, *p* < 0.001) (Figure 2B). Dehydrogenase activity showed remarkable enhancement in rotation treatments, with SZa increasing from 12.83 ± 0.76 to 21.53 ± 0.51 mg g^−1^ h^−1^ (*p* < 0.001) and SLa to 36.07 ± 0.90 mg g^−1^ h^−1^ (*p* < 0.001) compared to SSa (Figure 2C). Conversely, sucrase activity was significantly reduced across all rotation and fallow treatments. SZa decreased from 34.17 ± 0.90 to 23.42 ± 1.00 mg g^−1^ h^−1^ (*p* < 0.001) and SLa to 26.99 ± 1.84 mg g^−1^ h^−1^ (*p* = 0.003) compared to SSa, while SZAFa reduced from 23.31 ± 0.43 to 19.09 ± 0.70 mg g^−1^ h^−1^ (*p* = 0.006) and SLAFa to 17.80 ± 0.33 mg g^−1^ h^−1^ (*p* < 0.001) compared to SSAFa (Figure 2D).

**Figure 3:**
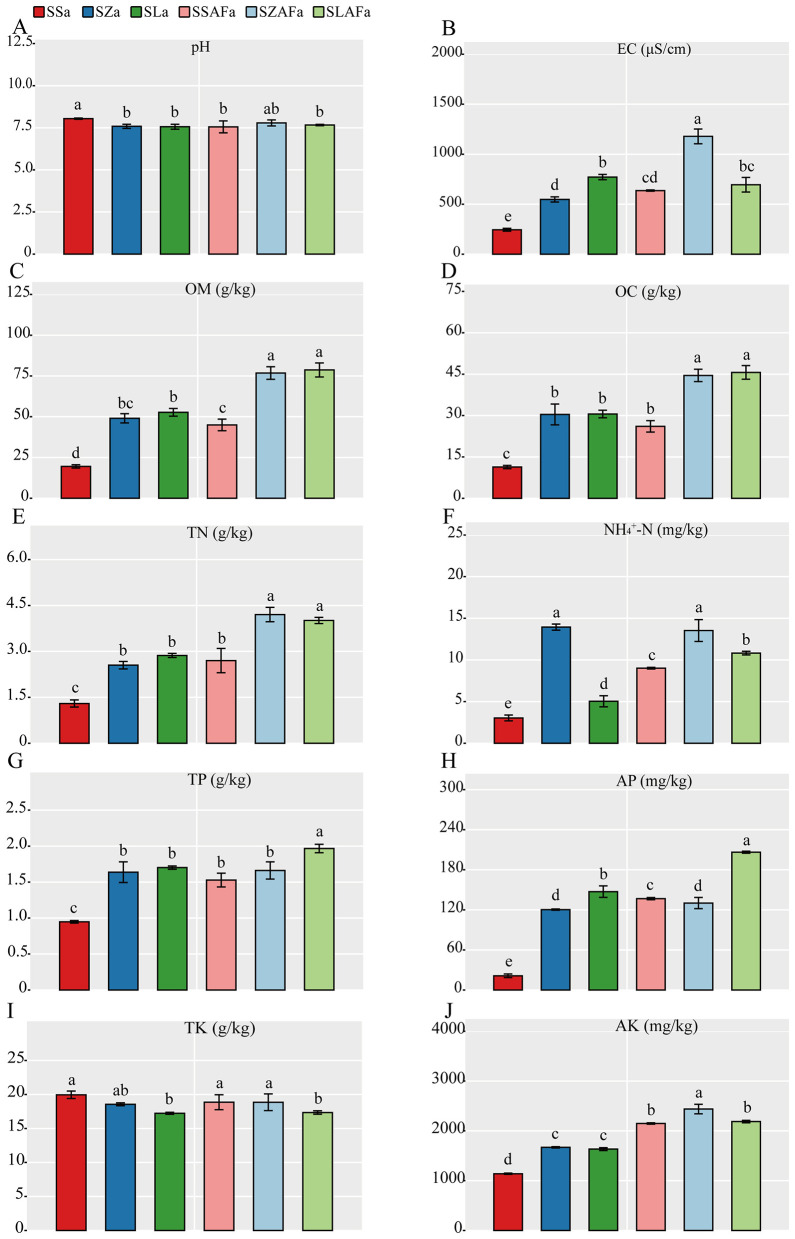
Soil pH **(A)**, electrical conductivity (EC) **(B)**, organic matter (OM) **(C)**, organic carbon (OC) **(D)**, total nitrogen (TN) **(E)**, ammonium nitrogen (${\text{NH}}_{4}^{+}$-N) **(F)**, total phosphorus (TP) **(G)**, available phosphorus (AP) **(H)**, total potassium (TK) **(I)**, and available potassium (AK) **(J)** under different crop rotation patterns.

Nutrient analysis revealed that both SZa and SLa treatments significantly reduced soil pH compared to SSa, decreasing from 8.04 ± 0.04 to 7.58 ± 0.10 (*p* = 0.002) and 7.56 ± 0.10 (*p* = 0.001), respectively (Figure 3A). Concurrently, electrical conductivity increased markedly from 244.6 ± 14.1 μS cm^−1^ to 553.0 ± 25.8 μS cm^−1^ (*p* < 0.001) and 771.5 ± 20.2 μS cm^−1^ (*p* < 0.001) for SZa and SLa, respectively (Figure 3B). This increasing trend in electrical conductivity persisted following fallow treatment, with SZAFa and SLAFa showing elevations from 636.2 ± 6.1 μS cm^−1^ to 1178.4 ± 73.4 μS cm^−1^ (*p* = 0.003) and 632.0 ± 71.0 μS cm^−1^ (*p* = 0.004) compared to SSAFa.

Both rotation treatments significantly enhanced the contents of several key nutrients relative to SSa. Organic matter increased from 19.6 ± 1.0 g kg^−1^ to 46.2 ± 2.9 g kg^−1^ (*p* < 0.001) and 50.2 ± 2.3 g kg^−1^ (*p* < 0.001) for SZa and SLa, respectively. Similarly, organic carbon rose from 11.3 ± 0.6 g kg^−1^ to 26.8 ± 2.9 g kg^−1^ (*p* < 0.001) and 29.1 ± 1.4 g kg^−1^ (*p* < 0.001). Total nitrogen increased from 1.3 ± 0.1 g kg^−1^ to 2.6 ± 0.1 g kg^−1^ (*p* < 0.001) and 2.9 ± 0.1 g kg^−1^ (*p* < 0.001). Ammonium nitrogen showed substantial increases from 3.0 ± 0.4 mg kg^−1^ to 13.9 ± 0.5 mg kg^−1^ (*p* < 0.001) and 5.0 ± 0.3 mg kg^−1^ (*p* = 0.002). Total phosphorus rose from 0.9 ± 0.0 g kg^−1^ to 1.6 ± 0.1 g kg^−1^ (*p* < 0.001) and 1.7 ± 0.0 g kg^−1^ (*p* < 0.001), while available phosphorus increased dramatically from 21.3 ± 2.3 mg kg^−1^ to 120.4 ± 0.5 mg kg^−1^ (*p* < 0.001) and 142.3 ± 7.6 mg kg^−1^ (*p* < 0.001).

The incorporation of fallow practices maintained nutrient accumulation effects in most parameters. Compared to SSAFa, SLAFa treatment significantly increased organic matter to 78.9 ± 2.1 g kg^−1^ (*p* = 0.001), organic carbon to 45.6 ± 1.9 g kg^−1^ (*p* = 0.002), total nitrogen to 4.0 ± 0.1 g kg^−1^ (*p* = 0.008), ammonium nitrogen to 10.6 ± 0.2 mg kg^−1^ (*p* = 0.021), and total phosphorus to 2.0 ± 0.0 g kg^−1^ (*p* = 0.003), while SZAFa showed no significant differences in these parameters.

Notably, SZAFa treatment reduced available phosphorus from 137.4 ± 1.4 mg kg^−1^ to 130.2 ± 8.4 mg kg^−1^ (*p* = 0.048), whereas SLAFa treatment increased it to 206.3 ± 1.6 mg kg^−1^ (*p* < 0.001). SLa treatment significantly decreased total potassium from 20.3 ± 0.5 g kg^−1^ to 17.1 ± 0.4 g kg^−1^ (*p* = 0.012), an effect that remained significant after fallow treatment, with SLAFa reducing it from 18.2 ± 1.0 g kg^−1^ to 17.6 ± 0.4 g kg^−1^ (*p* = 0.042) compared to SSAFa. Finally, SZAFa treatment significantly enhanced available potassium content from 2,148 ± 15 mg kg^−1^ to 2,437 ± 95 mg kg^−1^ (*p* = 0.009; Figure 3).

### 3.2 Soil microbial community diversity

**Figure 4:**
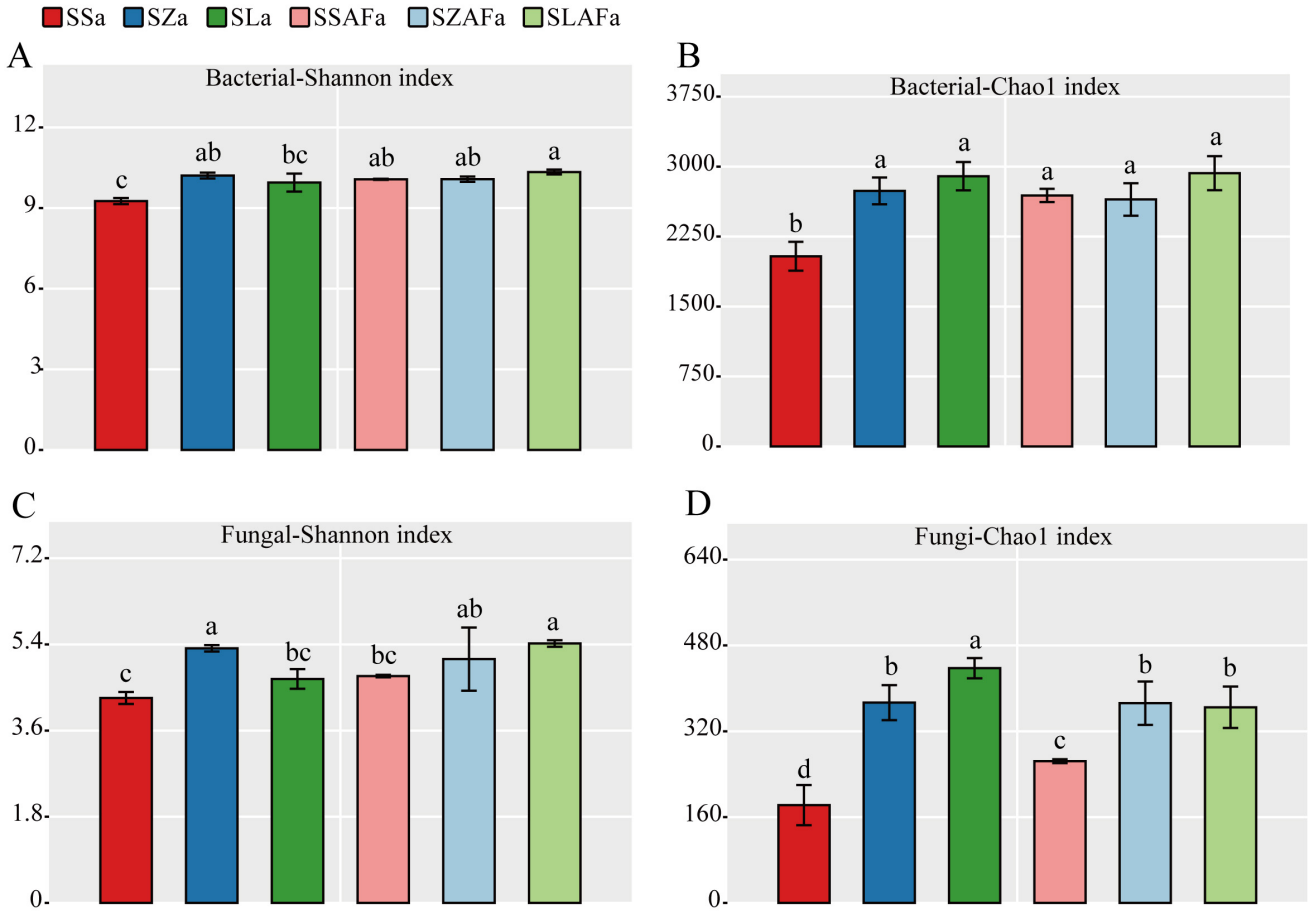
Bacterial diversity indices **(A, B)** and fungal diversity indices **(C, D)** in soil under different crop rotation patterns.

Different rotation systems significantly regulated soil microbial community diversity and structure. Compared with SSa, the SZa treatment increased the bacterial Shannon index from 9.26 ± 0.13 to 10.20 ± 0.10 (*p* = 0.003; Figure 4A). Both SZa and SLa treatments enhanced the bacterial Chao1 index, increasing from 1,971 ± 114 to 2754 ± 101 (*p* < 0.001) and 2,830 ± 134 (*p* < 0.001), respectively (Figure 4B). For the fungal community, SZa elevated the Shannon index from 4.16 ± 0.13 to 5.32 ± 0.06 (*p* < 0.001), while SLAFa increased it from 4.75 ± 0.02 to 5.47 ± 0.03 (*p* = 0.002) compared to SSAFa (Figure 4C). Both SZa and SLa treatments significantly improved the fungal Chao1 index from 166.7 ± 34.8 to 384.1 ± 33.5 (*p* = 0.002) and 459.3 ± 23.1 (*p* < 0.001), respectively. Similarly, SZAFa and SLAFa treatments promoted its increase compared to SSAFa, rising from 265.5 ± 5.0 to 325.7 ± 12.1 (*p* = 0.011) and 327.0 ± 10.2 (*p* = 0.009), respectively (Figure 4D).

**Figure 5:**
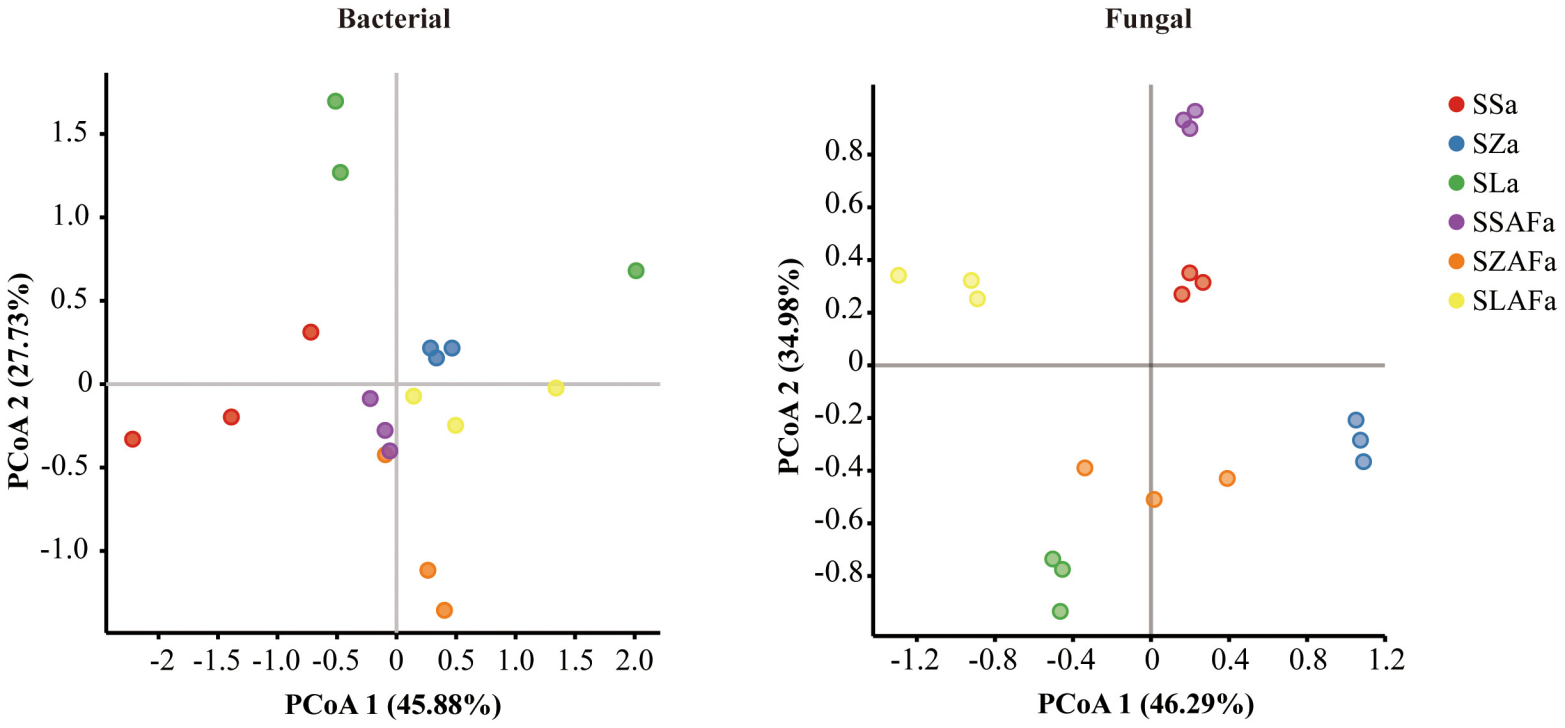
Principal coordinate analysis (PCoA) of soil microbial communities based on weighted UniFrac distances.

Principal coordinate analysis (PCoA) based on weighted UniFrac distances visually revealed distinct separation trends in bacterial and fungal community structures under different rotation treatments (Figure 5). To verify the statistical significance of these inter-group differences, we further performed PERMANOVA analysis. The results demonstrated that rotation treatments significantly altered both bacterial (*R*^2^ = 0.560, *p* = 0.001) and fungal (*R*^2^ = 0.576, *p* = 0.001) community structures, explaining 56.0% and 57.6% of the community variation, respectively. This statistical evidence confirms that different rotation systems have a restructuring effect on soil microbial communities.

At the phylum level, the dominant bacterial taxa included Actinobacteriota (37.7%), Proteobacteria (17.3%), Crenarchaeota (7.6%), Chloroflexi (11.3%), and Firmicutes (9.2%), collectively accounting for 83.1% of the total abundance. Both SZa and SLa treatments significantly reduced the abundance of Actinobacteriota (by 21.3%−56.0%), though this phylum remained the absolute dominant group (Supplementary Figure S2A). At the class level, Actinobacteria (43.9%−54.2%) was the predominant taxon. At the order level, Micromonosporales (9.4%−18.2%), Micrococcales (7.0%−29.2%), and Propionibacteriales (8.6%−21.4%) were identified as the dominant groups (Supplementary Figure S2B). SZa and SLa treatments significantly increased the abundance of Micromonosporales while significantly reducing the abundances of Micrococcales and Propionibacteriales (Supplementary Figure S2C).

For the fungal community, Ascomycota (80.9%−90.1%) was the overwhelmingly dominant phylum. Its abundance was significantly reduced by SZa and SLa compared to SSa, and a similar trend was observed for SZAFa and SLAFa compared to SSAFa (Supplementary Figure S3A). At the class level, Sordariomycetes (36.0%−58.6%) was the dominant group, and its abundance was significantly increased by SZa and SLa treatments (Supplementary Figure S3B). Order-level analysis revealed Hypocreales (10.0%−53.3%) and Microascales (2.0%−53.3%) as the major dominant taxa (Supplementary Figure S3C). SZa and SLa treatments significantly reduced the abundance of Hypocreales while significantly increasing that of Microascales. Similarly, SZAFa and SLAFa significantly suppressed the abundance of Hypocreales compared to SSAFa (Supplementary Figure S3D).

A heatmap analysis of the top 20 genera across 18 soil samples demonstrated that different rotation and fallow practices significantly altered microbial abundance distributions (Supplementary Figures S4, S5). These results clarify that the rotation system reshapes the multi-level structural characteristics of microbial communities by regulating the abundance of key taxonomic units.

### 3.3 Soil microbial co-occurrence network analysis

Distinct rotation systems significantly altered the topological features of soil microbial co-occurrence networks. Compared with SSa, the SZa treatment markedly increased the number of nodes in the bacterial network, while SLa also raised the node count but reduced the number of edges. Relative to SSAFa, both SZAFa and SLAFa enhanced the number of edges and nodes in the bacterial network, indicating that crop rotation intensifies the complexity of bacterial interactions (Supplementary Figure S6).

In the fungal networks, SZa significantly increased both the number of edges and nodes compared to SSa, whereas SLa raised the node count but decreased the number of edges. When compared to SSAFa, both SZAFa and SLAFa substantially increased the number of edges and nodes in the fungal network, demonstrating that the rotation system exerts a reinforcing effect on fungal interaction networks (Supplementary Figure S7).

### 3.4 Soil chemical properties and microbial community relationships

**Figure 6:**
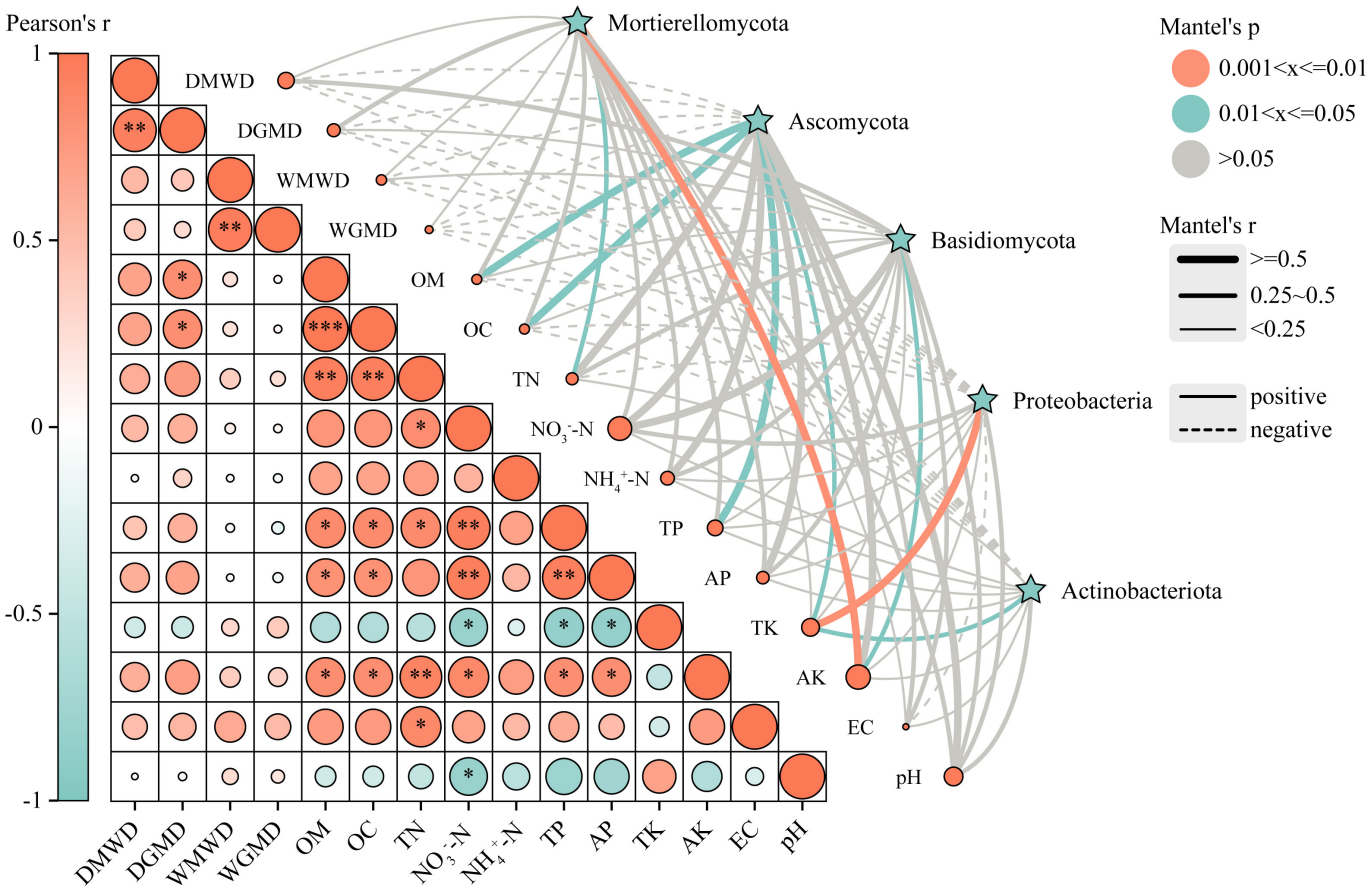
Mantel test correlation between soil aggregate structure, soil chemical properties, and microbial communities. DMWD, dry-sieved mean weight diameter; DGMD, dry-sieved geometric mean particle size; WMWD, wet-sieved mean weight diameter; WGMD, wet-sieved geometric mean; pH, particle size; EC, electrical conductivity; OM, organic matter; OC, organic carbon; TN, total nitrogen; ${\text{NH}}_{4}^{+}$-N, ammonium nitrogen; ${\text{NO}}_{3}^{-}$N, nitrate nitrogen; TP, total phosphorus; AP, available phosphorus; TK, total potassium; AK, available potassium. The statistical significance of the differences between groups is marked with asterisks: *p < 0.05, **p < 0.01, ***p < 0.001.

Mantel test analysis demonstrated significant correlations between soil microbial communities and multiple chemical properties (Figure 6). Specifically, Actinobacteriota exhibited a significant positive correlation with total potassium (TK) content (0.25 > Mantel's *r* > 0; 0.05 > *p* > 0.01), as did Proteobacteria (Mantel's *r* ≤ 0.5; 0.01 > *p* > 0.001). Basidiomycota showed a significant positive correlation with available potassium (AK) content (0.25 > Mantel's *r* > 0; 0.05 > *p* > 0.01). Ascomycota was significantly positively correlated with organic matter (OM), organic carbon (OC), total phosphorus (TP) (Mantel's *r* ≤ 0.5; 0.05 > *p* > 0.01), and TK content (0.25 > Mantel's *r* > 0; 0.05 > *p* > 0.01). Mortierellomycota demonstrated significant positive correlations with total nitrogen (TN) (0.25 > Mantel's *r* > 0; 0.05 > *p* > 0.01) and exhibited an even stronger correlation with AK content (Mantel's *r* ≤ 0.5; 0.01 > *p* > 0.001). These findings indicate that specific microbial taxa (e.g., Actinobacteriota, Proteobacteria) are significantly associated with key soil nutrient indicators (TK, AK, OM, etc.), revealing functional linkages between microbial community structure and soil chemical properties.

## 4 Discussion

### 4.1 Dynamics of soil chemical properties and enzyme activities

The rotation systems significantly improved soil physical structure through distinct mechanistic pathways. It is well-established that maize roots secrete abundant organic compounds, which can serve as both microbial carbon sources and cementing agents for soil aggregation. The formation of macroaggregates is likely attributed to the stimulation of microbial activity by maize root exudates and the subsequent promotion of organic binding agents (Galloway et al., 2022; Wang et al., 2022). In contrast, under the seed pumpkin rotation treatment (SLa), the proportion of microaggregates significantly increased (Figures 1C, D). The stability of these microaggregates may benefit from polysaccharide substances secreted by the root system of this crop species. Specifically, seed pumpkin roots are known to exude abundant polysaccharides, which can act as effective biological binding agents to facilitate the formation and stabilization of microaggregates. The fallow practice further reduced the disruption of microaggregates caused by mechanical disturbances. These results not only validate the widely recognized role of deep-rooted maize in improving soil structure but also reveal a unique regulatory mechanism of seed pumpkin on microaggregate dynamics. The root exudates of seed pumpkin likely enhance aggregate stability through cementation effects—a phenomenon that, to our knowledge, has not been previously documented in the literature (Schäfer et al., 2022; Siddiqui et al., 2022).

The rotation systems significantly reduced soil pH while increasing electrical conductivity through biologically mediated processes. Maize and seed pumpkin root exudates, particularly organic acids, directly drove soil acidification while simultaneously dissolving minerals to release base cations (Ma et al., 2022; Dong et al., 2023). The parallel increases in available phosphorus content and phosphatase activity demonstrated microbial activation of phosphorus cycling (Skinuliene et al., 2022), with maize rotation exhibiting stronger phosphatase stimulation than seed pumpkin systems—likely attributable to maize-derived phenolic acids inducing phosphatase gene expression (Liu et al., 2016). The apparent paradox between decreased urease activity and elevated total nitrogen content suggests a shift toward organic nitrogen mineralization pathways, potentially through dehydrogenase-mediated processes that complement conventional urea hydrolysis (Elsharif et al., 2023).

The significant reduction in urease and sucrase activities observed in rotation treatments (SZa, SLa) compared to continuous cropping (SSa) (Figures 2B, D) likely represents not merely functional degradation but rather a strategic shift in microbial nutrient cycling. The decline in urease activity, a key enzyme in nitrogen cycling, may be attributed to altered nitrogen supply pathways: in long-term monoculture systems, deteriorating organic matter quality forces microbial communities to rely more heavily on simple nitrogen sources like urea, thereby maintaining elevated urease activity (Cui et al., 2023). In contrast, rotation crops (particularly maize) introduce root exudates and residues richer in structural organic compounds (e.g., cellulose, hemicellulose), stimulating microbial succession toward complex organic nitrogen mineralization (as evidenced by changes in Actinobacteriota relative abundance, Supplementary Figure S2) and consequently reducing dependence on rapid urea hydrolysis (Thiollet-Scholtus et al., 2020).

Similarly, the decreased sucrase activity suggests a transformation in microbial carbon utilization strategies. Sucrose, as an easily available carbon source, tends to be preferentially utilized by pathogens or specific microbial communities in continuous cropping systems (Liu et al., 2023). The introduction of novel organic matter through rotation promotes the development of microbial taxa capable of decomposing complex carbon sources (e.g., fibers and lignin), shifting the overall metabolic function of the microbial community from simple carbon utilization to complex organic matter decomposition (Zhang et al., 2020). This metabolic transition results in reduced sucrase activity (Niu et al., 2024). Collectively, the decline in these enzyme activities may indicate a microbial strategy shift from “rapid nutrient cycling” to “steady-state nutrient cycling,” potentially serving as a biomarker for enhanced soil ecosystem health and stability (Qin et al., 2022).

### 4.2 Microbial-mediated improvement of soil structure

The rotation system exerts differential effects on the soil microbial community and physical structure, with close interactions observed between these two components. The restructuring of microbial diversity and functional groups likely serves as a key driver for soil structure improvement (Ali et al., 2019). Rotation practices provide diverse root exudates and residues (e.g., cellulose from maize straw), and this heterogeneous carbon input promotes the proliferation and succession of bacterial and fungal communities (Figure 4, Supplementary Figures S2, S3). Specifically, bacteria with polysaccharide-secreting capabilities (such as certain Proteobacteria and Actinobacteria species) and fungi that form extensive mycelial networks contribute to soil aggregation through mechanical entanglement of soil particles and the secretion of viscous extracellular polymeric substances (EPS). These processes directly facilitate the cementation of microaggregates into macroaggregates, explaining the shifts in aggregate size distribution observed in maize and pumpkin rotation treatments (Lybrand et al., 2022).

On the other hand, functional shifts in microbial communities also indirectly promote aggregate formation. Increased phosphatase activity under rotation (Figure 2A) reflects enhanced phosphorus cycling and microbial metabolism, while decreased sucrase activity indicates a microbial transition from utilizing simple carbon sources to decomposing complex organic matter (Tasswar et al., 2023; Wang et al., 2023). This process is accompanied by the synthesis of stable organic compounds (e.g., humic substances), which serve as long-term binding agents for the formation of water-stable aggregates. Thus, rotation practices foster a more diverse and functional microbial network, which collectively enhances aggregate formation and stability through both biophysical and biochemical pathways, ultimately improving soil physical structure and creating a healthier growth environment for crop roots.

### 4.3 Restructuring of microbial community diversity and functional groups

The rotation systems significantly enhanced α-diversity in both bacterial and fungal communities, primarily driven by the introduction of heterogeneous carbon sources through diversified cropping (Schmidt et al., 2018). Maize stover provided cellulose-rich substrates (Huang et al., 2017), while seed pumpkin root exudates released phenolic compounds (Zhang et al., 2022), collectively disrupting the dominance of Actinobacteriota in monoculture soils. Proteobacteria maintained stable abundance due to their versatile metabolic capabilities (Gu et al., 2024). Fungal communities exhibited more complex responses, with reduced Ascomycota abundance reflecting interrupted pathogen life cycles and increased Sordariomycetes abundance likely associated with maize residue decomposition (Xia et al., 2023). The decline in Hypocreales populations demonstrated the systems' pathogen-suppressive effects.

### 4.4 Ecological function enhancement in microbial co-occurrence networks

The rotation systems significantly enhanced microbial interaction network complexity through taxon-specific mechanisms. In bacterial networks, Proteobacteria utilized labile carbon sources for rapid growth while Actinobacteriota shifted toward recalcitrant organic matter decomposition (Liu et al., 2022b), forming mutualistic relationships through resource partitioning—a pattern further intensified by fallow practices. Fungal networks exhibited more pronounced restructuring, with simultaneous reduction in pathogenic nodes and strengthened saprophytic interactions. Maize rotation demonstrated superior optimization of bacterial networks compared to seed pumpkin systems, likely due to its extensive root biomass generating broader carbon source gradients (Yang et al., 2016). Conversely, seed pumpkin rotation uniquely activated phosphorus-cycling fungi, demonstrating specialized fungal network modulation. These findings reveal distinct crop-specific regulation mechanisms for bacterial vs. fungal networks, providing a theoretical foundation for designing functionally complementary rotations—maize to enhance bacterial interactions and seed pumpkin to stimulate fungal functionalities.

### 4.5 Integrated mechanisms of soil chemical-microbial interactions

Mantel tests revealed significant microbe-nutrient linkages, with distinct functional mechanisms identified across microbial taxa. Actinobacteriota likely contribute to potassium mineralization through organic acid secretion (Si et al., 2022), while Proteobacteria enhance available potassium release via specialized metabolic pathways (Bian et al., 2023). The positive correlations between Ascomycota and both organic matter and total phosphorus reflect their decomposition capabilities, though their reduced abundance under rotation indicates optimized carbon-phosphorus cycling efficiency. Seed pumpkin rotation significantly decreased total potassium content, potentially due to disequilibrium between crop uptake and microbial-mediated potassium mobilization. This crop-specific potassium demand dynamic provides new insights for precision nutrient management in diversified cropping systems.

### 4.6 Limitations of the study

This study has several limitations that should be acknowledged. The sample size of *n* = 3 per group, as indicated by a *post hoc* power analysis, provides approximately 70% power to detect large effects (*d* = 1.2), potentially overlooking small yet ecologically meaningful differences. Future research should increase replicates to at least *n* = 5 or conduct long-term fixed-location trials to enhance statistical robustness. Additionally, only one rotation season was observed, which limits the ability to evaluate the interannual stability and cumulative effects of the treatments. Finally, as the experiment was conducted solely in an oasis irrigated region of Xinjiang, the generalizability of the findings to other climatic and edaphic contexts remains uncertain and requires further validation.

## 5 Conclusion

This study systematically elucidates the soil microecological regulation mechanisms in maize-seed pumpkin-processing tomato rotation systems. Maize optimizes soil physical structure through deep root system effects while simultaneously enhancing bacterial network functionality. Seed pumpkin activates phosphorus cycling processes via root exudates and restructures fungal community composition. The rotation systems effectively mitigate continuous cropping obstacles, though particular attention must be paid to dynamic potassium balance to prevent fertility imbalances characteristic of monoculture systems. Rotation design should integrate crop-specific nutrient demand traits to achieve balanced soil nutrient dynamics.

These findings establish a theoretical foundation for optimizing processing tomato production systems in Xinjiang. The functional complementarity between maize and seed pumpkin warrants deeper exploration, necessitating long-term field trials to evaluate ecological and economic benefits of different rotation combinations. Such trials should prioritize developing reliable practical guidelines through continuous monitoring of system performance, ultimately enabling refinement of nutrient management strategies to advance sustainable agricultural intensification.

## Data Availability

The datasets used in this study have been deposited in an online repository. The repository link (https://ngdc.cncb.ac.cn/) and corresponding accession number (PRJCA047176) are provided herein for access.
