# Supplementary material for: Differential regulation of soil microecology in crop rotation systems of maize, seed pumpkin, and processing tomato
Source: Front Microbiol. 2025 Nov 17;16:1640980. doi: 10.3389/fmicb.2025.1640980 (PMC12665708; doi:10.3389/fmicb.2025.1640980)
Supplement: Supplementary file 1 [file Data_Sheet_1.docx]

Supplementary Material

# Supplementary Figures and Tables

## Supplementary Figures


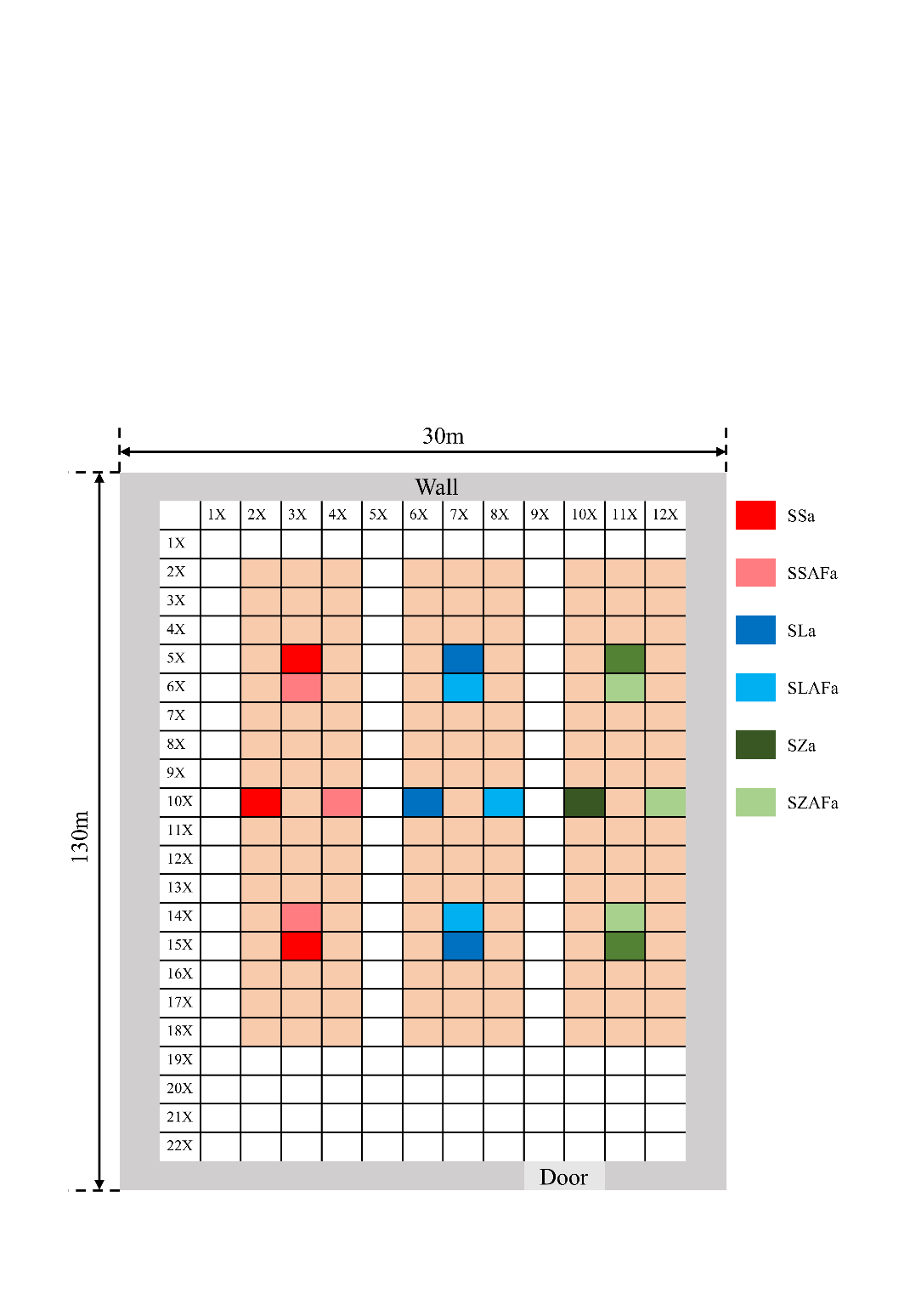


Supplementary Figure S1. Schematic diagram of the layout of the experimental field and the collection of soil samples


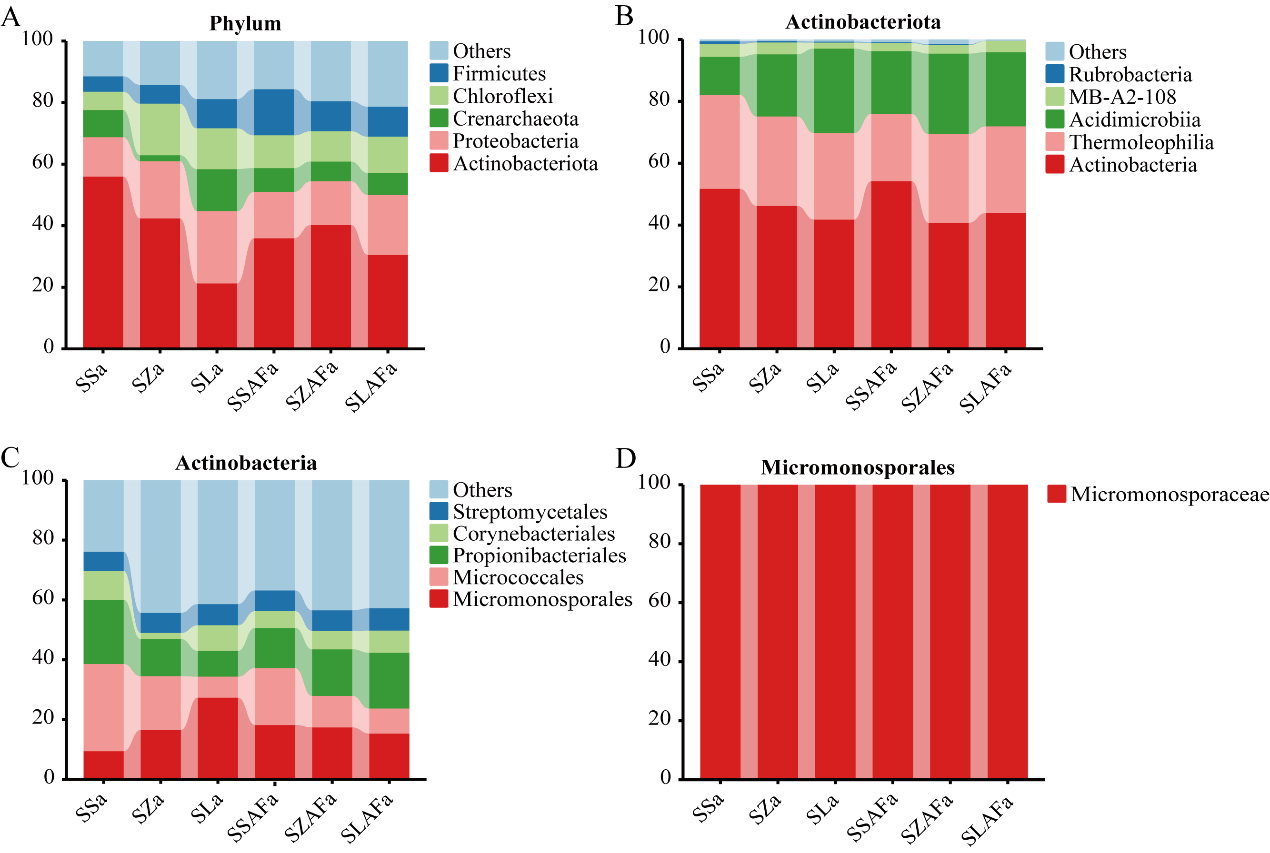


Supplementary Figure S2. The relative abundance of soil bacterial communities under different crop rotation patterns.


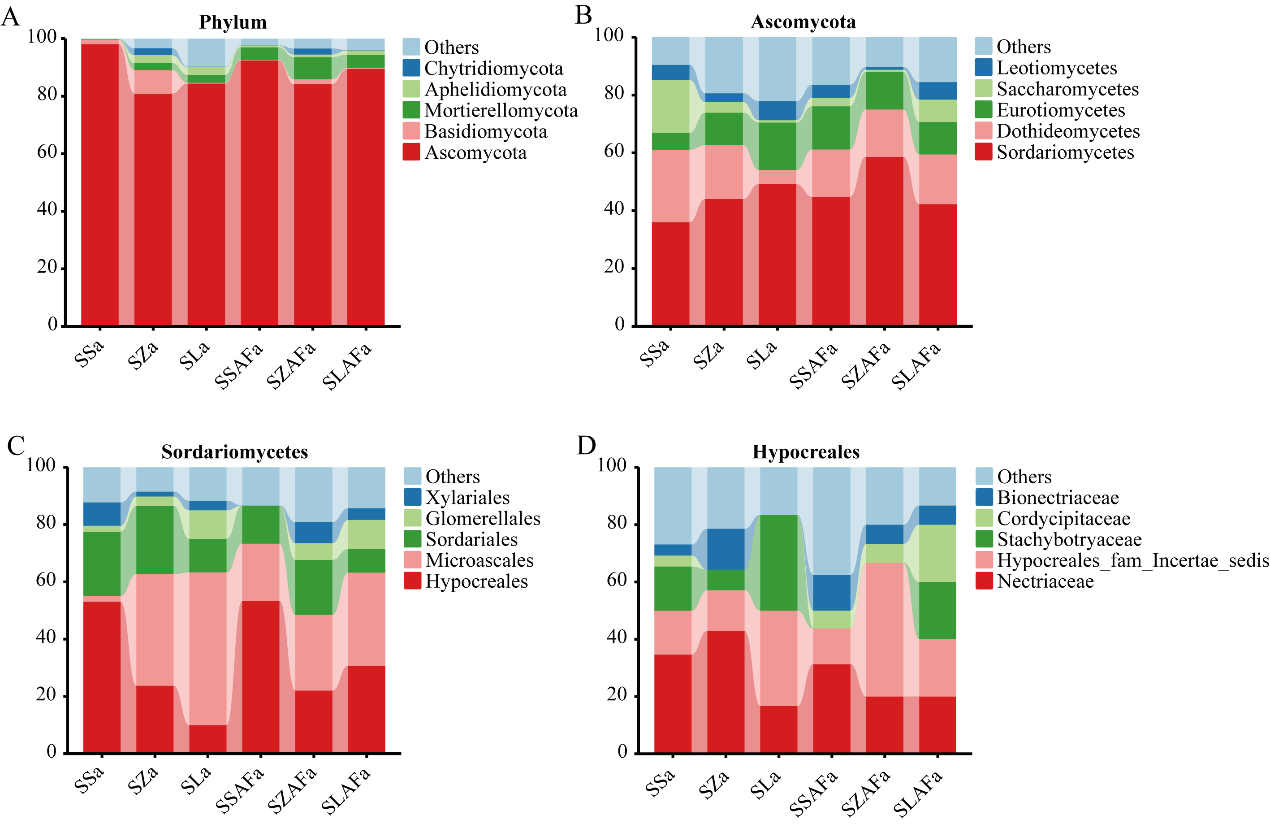


Supplementary Figure S3. The relative abundance of soil fungal communities under different crop rotation patterns.


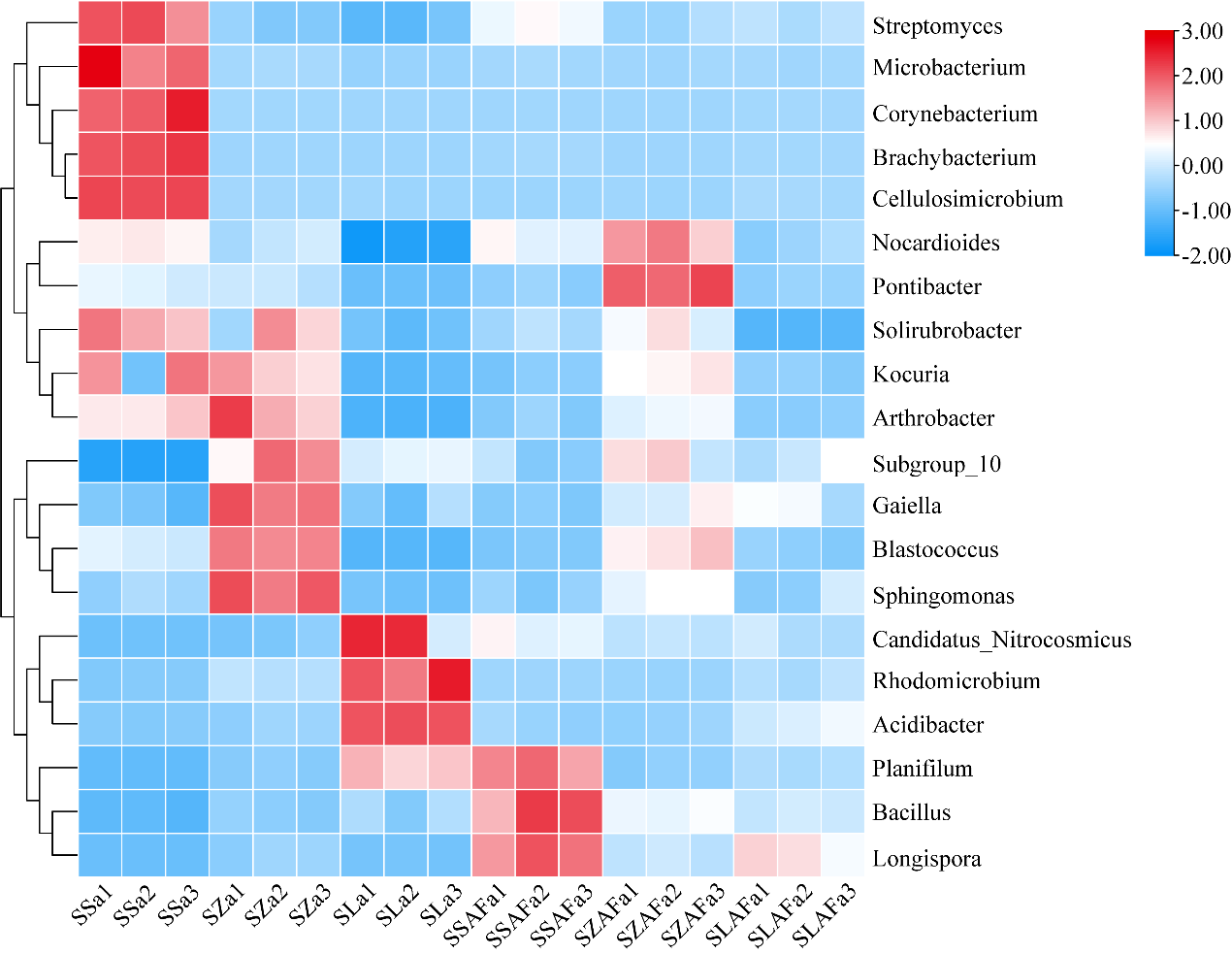


Supplementary Figure S4. Horizontal heat map analysis of soil bacterial communities under different crop rotation modes.


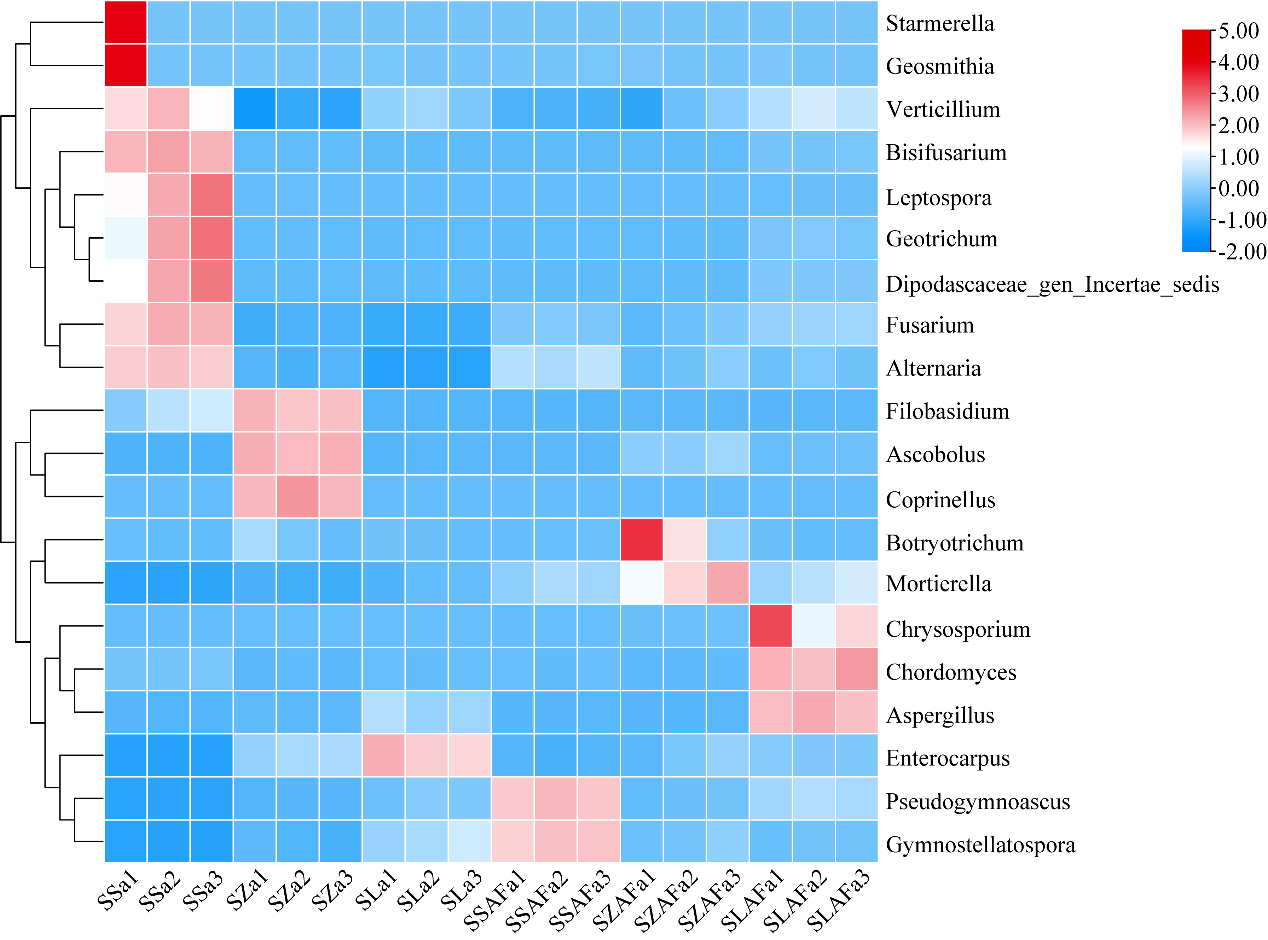


Supplementary Figure S5. Horizontal heat map analysis of soil fungal communities under different crop rotation patterns.


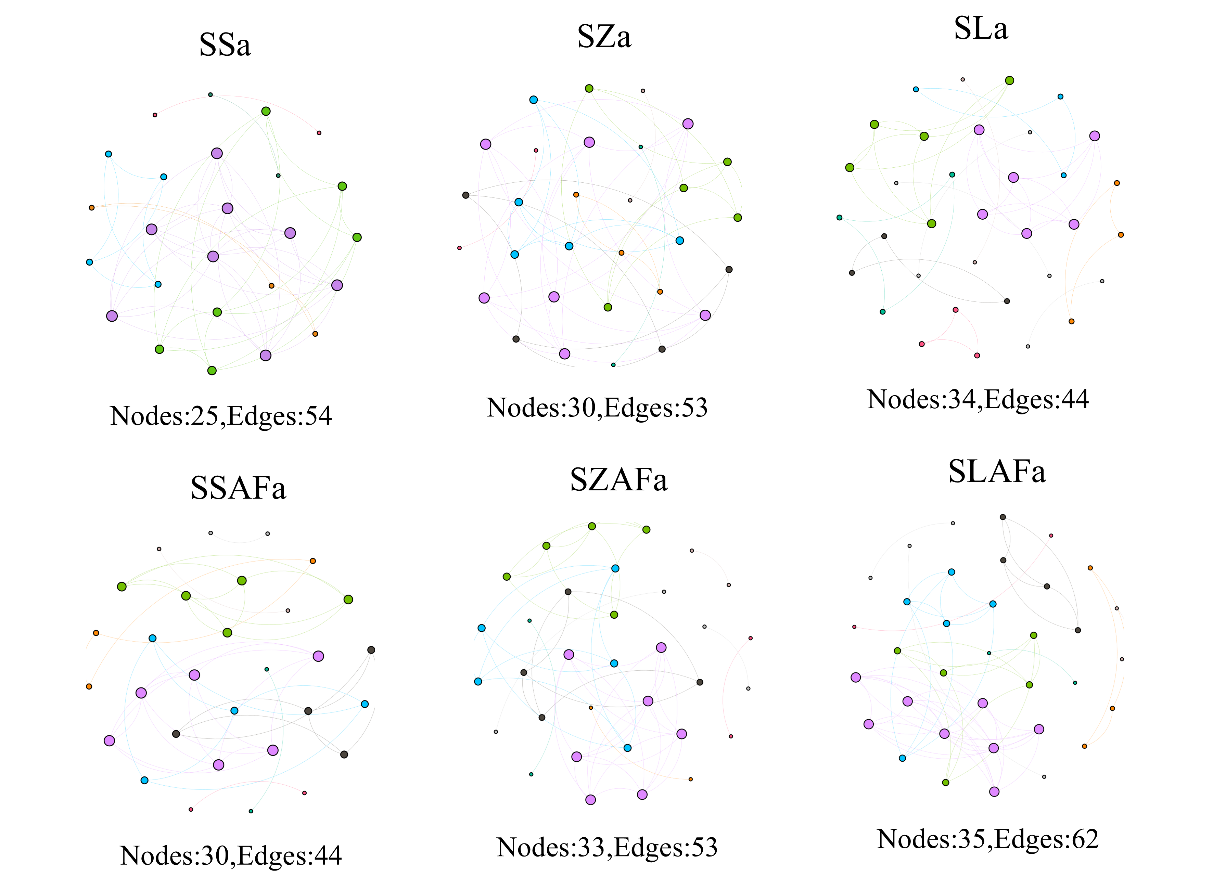


Supplementary Figure S6. Soil bacteria co-occurrence network diagrams under different crop rotation modes. The network was constructed based on Spearman correlation (ρ > 0.6, FDR corrected p ≤ 0.05). The size of the node is proportional to the degree centrality, and the color represents different modules (detected by the Fast-Greedy algorithm).


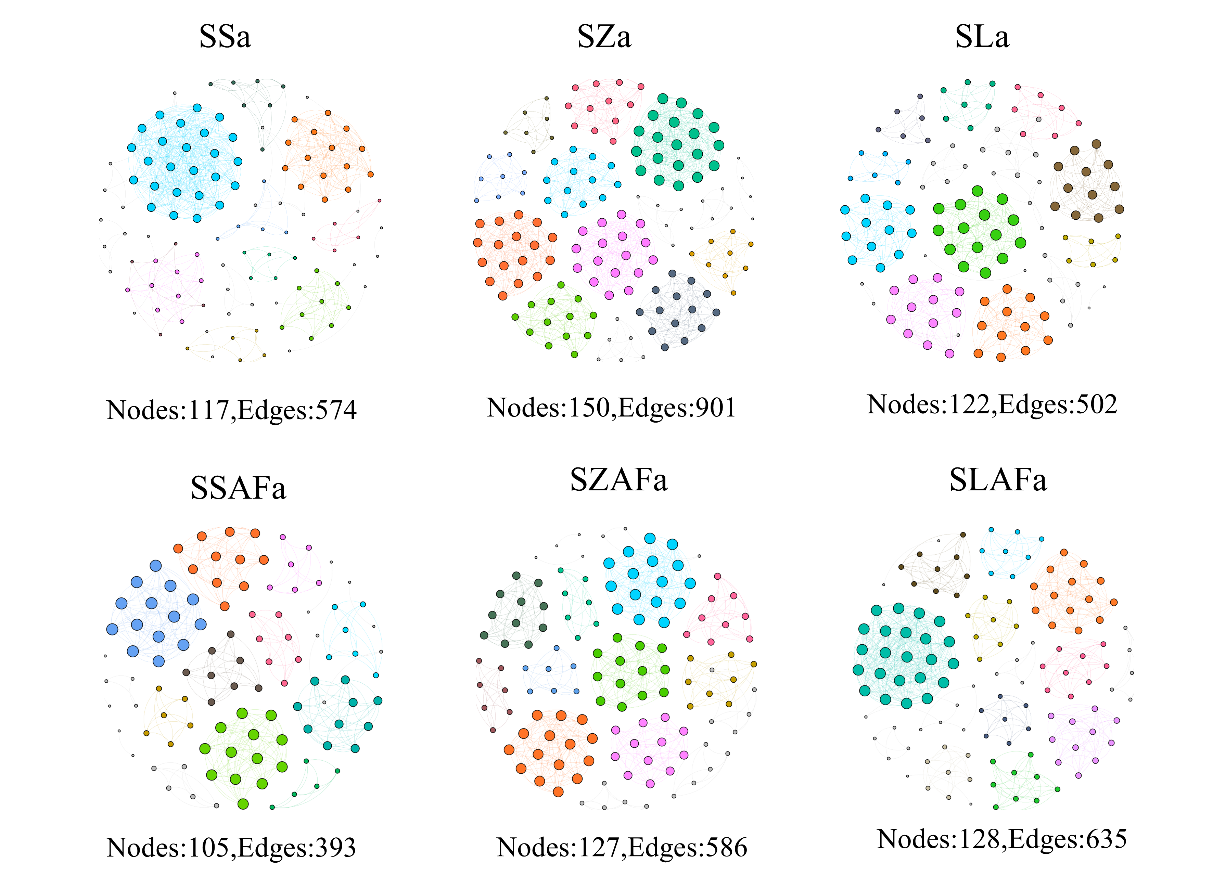


Supplementary Figure S7. Co-occurrence network diagrams of soil fungi under different crop rotation modes. The network was constructed based on Spearman correlation (ρ > 0.6, FDR corrected p ≤ 0.05). The size of the node is proportional to the degree centrality, and the color represents different modules (detected by the Fast-Greedy algorithm).


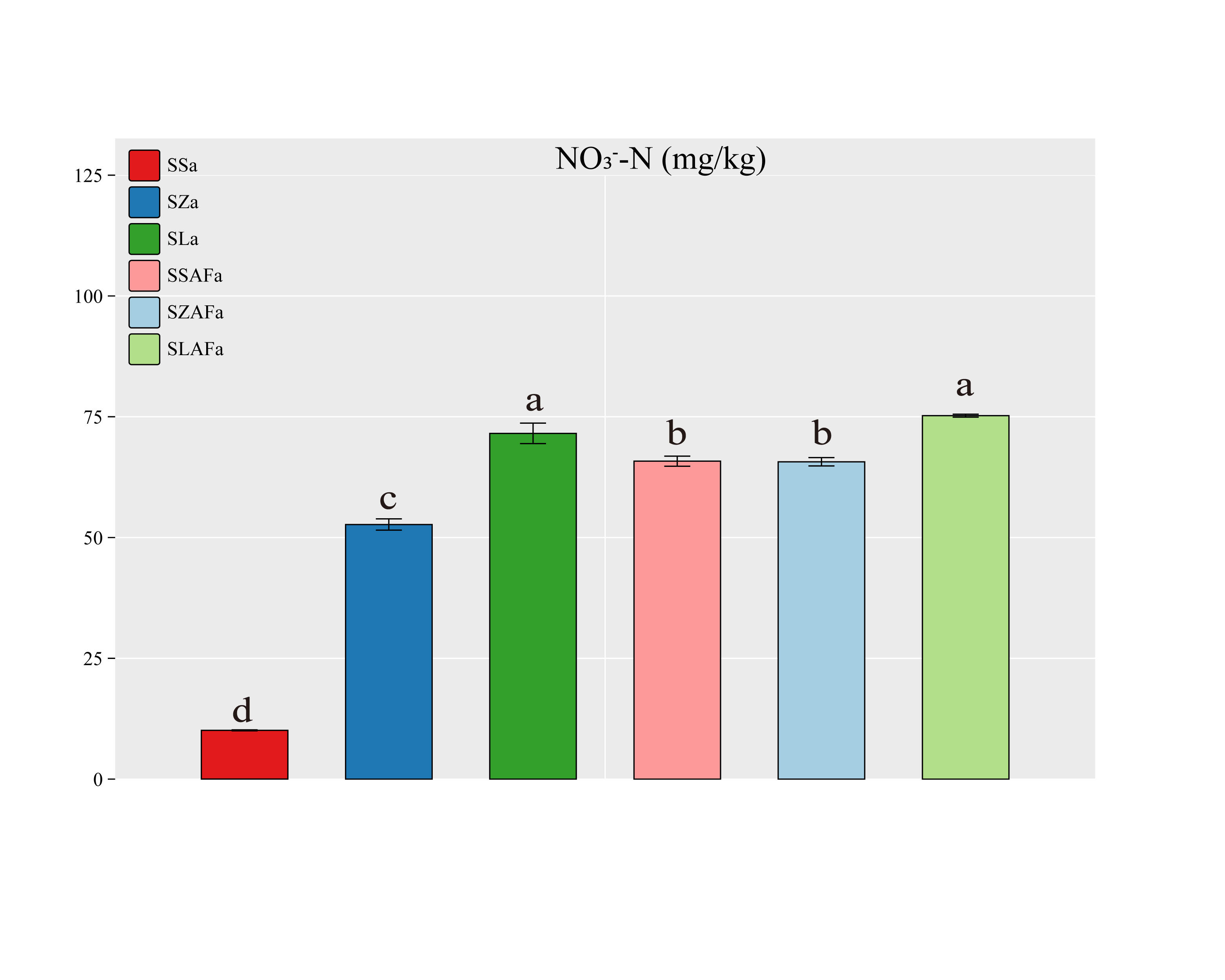


Supplementary Figure S8 Determination of Soil Nitrate Nitrogen Content.

Supplementary Table S1. Efficacy Analysis Table

| Domain | Index | Comparison | Cohen’s d | n | Power |
| --- | --- | --- | --- | --- | --- |
| Fungi | Chao1 | SSa vs SZa | 2.25 | 3 | 0.456 |
| Fungi | Chao1 | SSa vs SLa | 2.68 | 3 | 0.573 |
| Fungi | Chao1 | SSAFa vs SZAFa | 1.37 | 3 | 0.198 |
| Fungi | Chao1 | SSAFa vs SLAFa | 1.68 | 3 | 0.281 |
| Fungi | Shannon | SSa vs SZa | 4.95 | 3 | 0.94 |
| Fungi | Shannon | SSa vs SLa | 1.6 | 3 | 0.25 |
| Fungi | Shannon | SSAFa vs SZAFa | 1.28 | 3 | 0.178 |
| Fungi | Shannon | SSAFa vs SLAFa | 3.24 | 3 | 0.75 |
| Bacteria | Chao1 | SSa vs SZa | 3.78 | 3 | 0.81 |
| Bacteria | Chao1 | SSa vs SLa | 4.75 | 3 | 0.935 |
| Bacteria | Chao1 | SSAFa vs SZAFa | ~0 | 3 | 0.05 |
| Bacteria | Chao1 | SSAFa vs SLAFa | 1.32 | 3 | 0.208 |
| Bacteria | Shannon | SSa vs SZa | 6.33 | 3 | 0.989 |
| Bacteria | Shannon | SSa vs SLa | 5.1 | 3 | 0.95 |
| Bacteria | Shannon | SSAFa vs SZAFa | ~0 | 3 | 0.05 |
| Bacteria | Shannon | SSAFa vs SLAFa | 1.56 | 3 | 0.24 |
